# Increased *Enterococcus faecalis* infection is associated with clinically active Crohn disease

**DOI:** 10.1097/MD.0000000000005019

**Published:** 2016-09-30

**Authors:** Youlian Zhou, Huiting Chen, Hanchang He, Yanlei Du, Jiaqi Hu, Yingfei Li, Yuyuan Li, Yongjian Zhou, Hong Wang, Ye Chen, Yuqiang Nie

**Affiliations:** a Department of Gastroenterology, Guangzhou Digestive Disease Center, Guangzhou First People's Hospital, Guangzhou Medical University, Guangzhou; b The First People's Foshan Hospital, Chancheng District, Foshan, Guangdong; c Department of Pediatrics, Guangzhou First People's Hospital, Guangzhou Medical University; d Guangdong Provincial Key Laboratory of Gastroenterology, Department of Gastroenterology, Nanfang Hospital, Southern Medical University, Guangzhou, P. R. China.

**Keywords:** Crohn disease (CD), *Enterococcus faecalis*, *Fusobacterium* spp, ulcerative colitis (UC)

## Abstract

This study was performed to investigate the relationship between the abundance of pathogenic gut microbes in Chinese patients with inflammatory bowel disease (IBD) and disease severity.

We collected clinical data and fecal samples from 47 therapy-naive Chinese patients with ulcerative colitis (UC), 67 patients with Crohn disease (CD), and 48 healthy volunteers. Bacteria levels of *Fusobacterium* species (spp), enterotoxigenic *Bacteroides fragilis* (*B fragilis*), enteropathogenic *Escherichia coli* (*E coli*), and *Enterococcus faecalis* (*E faecalis*) were assessed by quantitative real-time PCR (qRT-PCR). Spearman correlation coefficients were calculated to test associations between bacterial content and clinical parameters.

Compared to healthy controls, the levels of both *Fusobacterium* spp and *E faecalis* were significantly increased in the feces of patients with IBD (*P* < 0.01). *B fragilis* levels were higher (*P* < 0.05) and *E faecalis* levels lower (*P* < 0.05) in patients with CD compared to those with UC. Increased *E faecalis* colonization in CD associated positively with disease activity (*P* = 0.015), Crohn disease activity index (CDAI; R = 0.3118, *P* = 0.0108), and fecal calprotectin (*P* = 0.016).

*E faecalis* and *Fusobacterium* spp are significantly enriched in patients with IBD, and increased *E faecalis* infection is associated with clinically active CD.

## 1 Introduction

Inflammatory bowel disease (IBD), comprising Crohn disease (CD) and ulcerative colitis (UC), is characterized by relapsing and remitting chronic inflammation of the gastrointestinal tract. The chronic course of recurrence during IBD progression gradually leads to complications, such as stricture, fistula formation, intestinal obstruction, intestinal perforation, toxic megacolon, and even cancer. Although only 1% to 2% of all colorectal cancer (CRC) cases are colitis-associated, CRC is considered a serious complication of IBD accounting for approximately 10% to 15% of all deaths in patients with IBD.^[1]^

The etiology and pathogenesis of IBD are not fully understood, but many clinical and experimental observations strongly implicate intestinal bacteria as significant contributors to disease initiation and progression.^[2,3]^ For example, antibiotics can ameliorate IBD symptoms, and genetically engineered IBD-susceptible rodents maintained under germ-free conditions are protected from IBD.^[4]^

Specific microorganisms directly associated with the pathogenesis of CD or UC have not been identified to date, but clinical and in vitro evidence suggests that the gut microbiome of patients with IBD differs significantly from that of healthy patients.^[5–8]^ Ott et al^[9]^ performed 16S ribosomal DNA (rDNA)-based single-strand conformation polymorphism fingerprinting to reveal that mucosal inflammation in IBD was associated with a loss of normal anaerobic bacteria. On the other hand, van der Waaij et al^[10]^ suggested that patients with active IBD or shortly after remission exhibited an increase in immunoglobulin-coated fecal anaerobic bacteria compared to healthy controls.

The ratio between different pathogenic and beneficial bacterial species is also altered in patients with IBD. *Pseudomonas* contributes to the pathogenesis of CD.^[6]^ Another study reported that the frequency of toxigenic *Clostridium difficile* was 7% higher in IBD patients than in healthy volunteers.^[11]^ Furthermore, an increased abundance in *Enterobacteriaceae*, *Pasteurellacaea*, *Veillonellaceae*, and *Fusobacteriaceae*, and decreased abundance in *Erysipelotrichales*, *Bacteroidales*, and *Clostridiales* were found to correlate strongly with disease status.^[12]^ Despite intensive research, the mechanisms by which bacteria affect the development of IBD or the disease-specific changes in the intestinal flora have not been determined to date. Thus, if the gut microbiome is considered to be a key driver of inflammation, the dysbiosis that precedes relapse could be a major therapeutic target.

Using microbial 16S ribosomal RNA (rRNA) sequencing, we previously showed that *Fusobacterium*, *Bacteroides*, *Enterococcus*, and *Streptococcus* were enriched in IBD patients (YZ and YC, unpublished observations). These species or genera have also been observed to be associated with CRC and its clinicopathological features.^[13–18]^ For example, multiple studies have demonstrated that fecal or tissue samples from CRC patients were enriched for specific bacterial pathogens, including *Fusobacterium*,^[13–15]^*Enterococcus faecalis* (*E faecalis*),^[16]^ enterotoxigenic *Bacteroides fragilis* (ETBF, *B fragilis*),^[19]^ enteropathogenic *Escherichia coli* (EPEC, *E coli*),^[17]^ and *Streptococcus gallolyticus*.^[20–22]^ Recently, Nakatsu et al^[23]^ also suggested that a taxonomically defined microbial consortium is implicated in the development of CRC. Furthermore, Ericsson et al^[24]^ identified a naturally occurring variation in gut microbes that was associated with CRC severity, and the abundance of certain taxa correlated with decreased tumor burden. Once we obtain a better understanding of the microbial dysbiosis underlying colorectal carcinogenesis, new strategies toward the diagnosis, treatment, and prevention of CRC may be realized.^[25]^

The objective of our study was to investigate the relationship between CRC-associated bacterial pathogens and IBD activity. We collected stool samples from Chinese patients presenting with CD or UC and healthy volunteers. We employed quantitative real-time polymerase chain reaction (qRT-PCR) to quantify the levels of four pathogens frequently associated with CRC, including *Fusobacterium* species (spp), ETBF, enteropathogenic *E coli*, and *E faecalis*. We then performed correlation analyses to determine whether the presence of these bacterial populations was associated with disease severity.

## 2 Materials and methods

### 2.1 Patients and samples

Patients with CD or UC who had not received any treatment for IBD were recruited between June 2012 and July 2013 at the Department of Gastroenterology of Nanfang Hospital, Southern Medical University, China. Healthy volunteers ranging in age from 20 to 40 years (to match the age and gender of patients with CD) were recruited from the general population around the Southern Medical University. Exclusion criteria included patients with previous IBD-treatment, receiving antibiotics or probiotics in the last 4 weeks, age <15 years, presentation of other known chronic diseases, and pregnancy or breast feeding. Fecal samples were collected from all enrolled subjects and stored at −80 °C before further processing.

All study protocols were in compliance with the Declaration of Helsinki and were approved by the Ethics Committee of both Nanfang Hospital, Southern Medical University and Guangzhou First People's Hospital affiliated with Guangzhou Medical University. Written consent for study participation was obtained from each volunteer.

### 2.2 IBD definitions and classification criteria

Diagnoses of UC and CD were based on the internationally accepted Lennard–Jones criteria.^[26]^ According to the Montreal classification, UC was categorized as ulcerative proctitis (E1), left-sided (distal) UC (E2), and extensive UC (pancolitis; E3), based on the extent of the disease.^[27]^ CD was classified based on location in the ileum (L1), colon (L2), or ileocolon (L3).^[27]^ For the evaluation of disease activity, the Mayo score^[28]^ for UC and Crohn disease activity index (CDAI) score^[29]^ for CD were determined (mild, S1; moderate, S2; or severe, S3).

### 2.3 Total bacterial genomic DNA extraction

Bacterial DNA was extracted from the fecal samples using the TIANamp Stool DNA Kit (TIANGEN Biotech, Beijing, China) according to the manufacturer's instructions.^[30]^ DNA concentrations were measured with a NanoDrop 2000 Bioanalyzer (Thermo Fisher Scientific, Inc., Waltham, MA). Samples were stored at −20 °C before qRT-PCR assays.

### 2.4 qRT-PCR

All primer sets for the 4 bacterial groups or species targeted the 16S rRNA gene and are listed in Table 1. 16S rRNA of each bacterial strain was cloned into the pUCm-T vector (Sangon, Shanghai, China) according to the manufacturer's procedure for use as a copy number standard. For each qRT-PCR standard, the copy number concentration was calculated based on the length of the PCR product and the average mass of a DNA base pair. The standards were stored at −80 °C, and serial dilutions (1–10^8^ copies/μL) were prepared prior to each qRT-PCR assay.^[35]^ Results for each sample were expressed as the copy number of bacterial 16S rDNA per gram of feces. Assays were performed in 96-well optical plates on the Light Cycler 480 Real-Time PCR System (Roche Diagnostics, Rotkreuz, Switzerland) in triplicate. The 20 μL reactions contained Light Cycler 480 SYBR Green I Master solution (Roche Diagnostics, Mannheim, Germany), the specific primer pairs at a final concentration of 0.5 μM, and 5 μL of DNA template. Amplifications were performed as follows: initial denaturation at 95 °C for 5 minutes, followed by 45 cycles of denaturation at 95 °C for 10 seconds, annealing at 52 to 56 °C (primer dependent) for 10 seconds, and extension at 72 °C for 10 seconds. The specificity of each amplification was assessed by melting curve analysis. The efficiency of amplification for each primer pair was estimated from the standard curves.

**Table 1:**
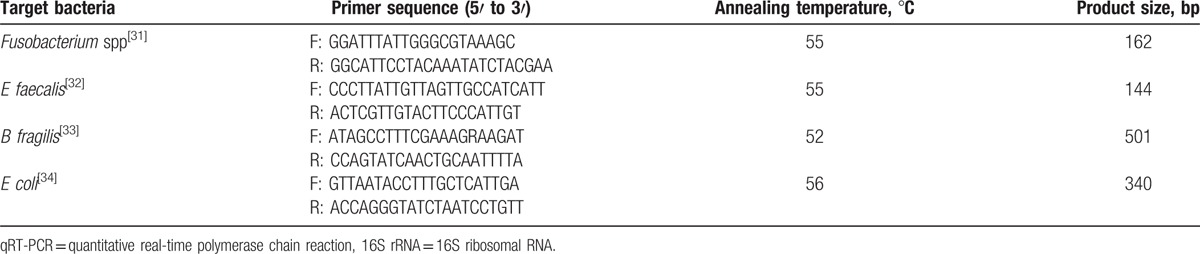
Primer sequences for pathogen-specific detection of 16S rRNA by qRT-PCR.

### 2.5 Fecal calprotectin (FC) assay

FC concentrations were measured with a quantitative PhiCal enzyme-linked immunosorbent assay kit (Immundiagnostik AG, Cat. No. K6927) according to the manufacturer's instructions. Fecal specimens were diluted 1:2500. Enzyme-linked immunosorbent assay plates were read by a Thermo Scientific Microplate Reader (Multiskan FC, optical density at 450 nm against 620 nm). Samples containing ≥100 μg of calprotectin per 1 g of feces were considered calprotectin-positive.

### 2.6 Statistical analysis

Data are presented as means ± standard error of the mean (SEM) for quantitative variables and as frequencies for qualitative variables. Given the nonnormal distribution of the data analyzed, a nonparametric test (Kruskal–Wallis test) was used to assess changes in bacterial number between groups. Results with a false discovery rate ≤0.05 after applying multiple test correction (Bonferroni correction method) for each species were considered significant. The Spearman correlation coefficient was calculated to estimate the correlations between variables. Statistical analyses were performed with the statistical software package SPSS16.0 (SPSS Inc., Chicago, IL). A 2-tailed *P* value of less than 0.05 was considered statistically significant.

## 3 Results

### 3.1 Patient characteristics

The clinical data for each patient cohort are summarized in Table 2. Of the 67 patients with CD (36 males and 31 females; mean age 31 ± 2 years), 60 presented with active disease and a mean CDAI index of 266.55 ± 13.099. CD in 11 patients was complicated by fistulizing disease. Of the 47 patients with UC (26 males and 21 females; mean age 42 ± 2.2 years), 46 presented with active disease and a mean Mayo score of 8.56 ± 0.396. Forty-eight healthy volunteers (23 males and 25 females; mean age 32.25 ± 0.97 years) who consumed primarily a traditional diet provided fresh fecal samples as controls. Neither age (*P* < 0.001) nor gender make-up (*P* = 0.746) different across these 3 groups.

**Table 2:**
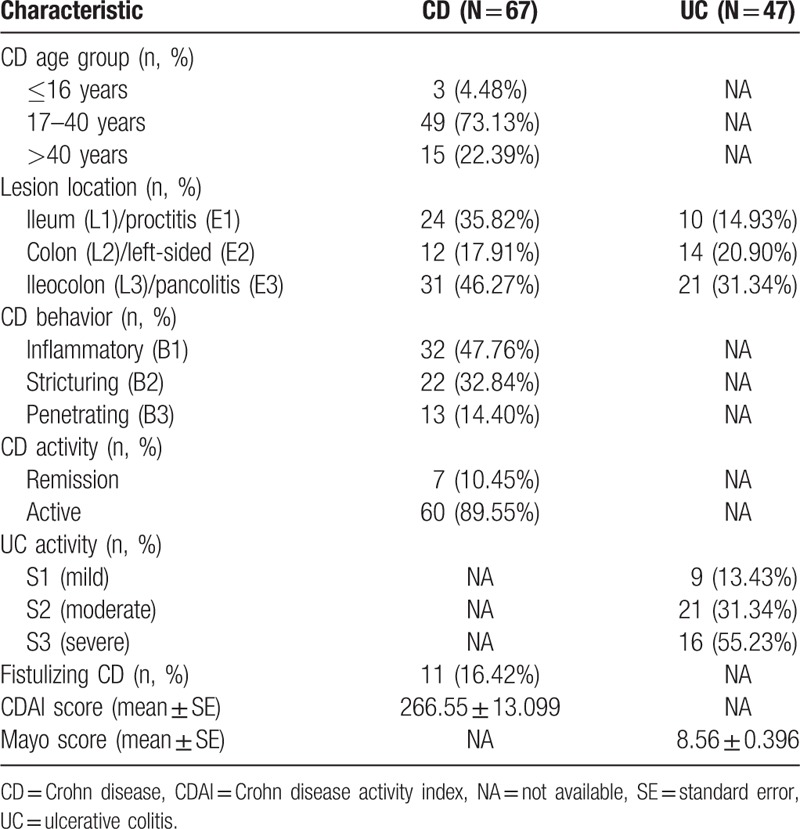
Baseline clinical characteristics of patients groups.

### 3.2 *Fusobacterium* spp and *E faecalis* are significantly increased in patients with IBD

**Figure 1:**
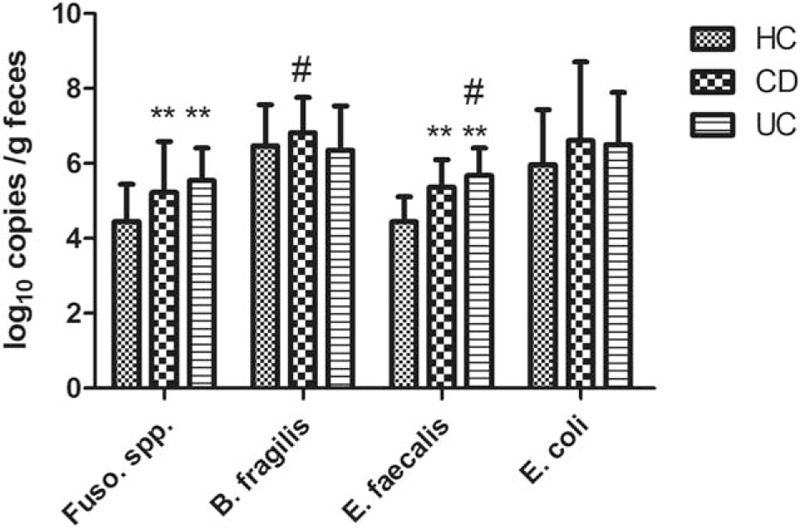
*Fusobacterium* spp and *E faecalis* are significantly increased in patients with IBD. Bacterial gene copy number (log_10_ copies/g feces) was determined by qRT-PCR based on 16S rRNA expression. Statistical significance was determined by the Kruskal–Wallis test followed by pairwise comparisons. ^∗∗^*P* < 0.01 versus healthy controls, ^#^*P* < 0.05 versus CD or UC. CD = Crohn disease, IBD = inflammatory bowel disease, qRT-PCR = quantitative real-time polymerase chain reaction, 16S rRNA = 16S ribosomal RNA, UC = ulcerative colitis.

We assessed the abundance of each bacterial species by measuring the expression of the 16S rRNA gene specific to each pathogen by qRT-PCR. The levels of *Fusobacterium* spp and *E faecalis* were significantly increased in the feces of patients with IBD (*P* < 0.01; Fig. 1). The levels of *B fragilis* were greater in patients with CD compared to those with UC (*P* < 0.05). Conversely, the levels of *E faecalis* were lower in patients with CD than in patients with UC (*P* < 0.05). No significant differences in the levels of *E coli* were detected between IBD patients and healthy controls (*P* > 0.05).

### 3.3 Increased *E faecalis* in CD is associated with disease activity

**Figure 2:**
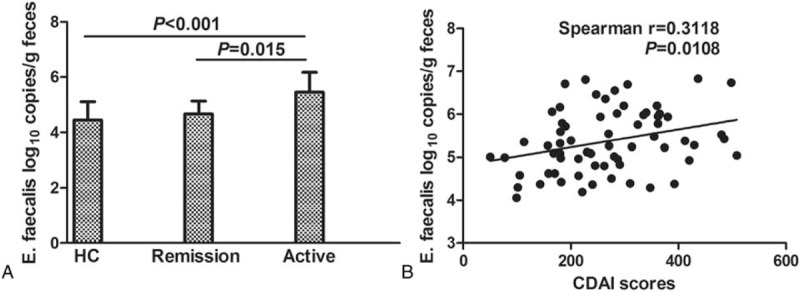
Increased *E faecalis* is associated with CD activity. (A) qRT-PCR results demonstrate the number of *E faecalis* gene copies (as log_10_ copies/g feces) based on 16S rRNA expression in HCs versus CD patients in remission or presenting with active disease. Statistical significance was evaluated by the Kruskal–Wallis test followed by all pairwise comparisons. (B) Correlation between *E faecalis* levels and CDAI score. A significant positive relationship was observed (Spearman correlation coefficient *R* = 0.3118, *P* = 0.0108). CD = Crohn disease, CDAI = Crohn disease activity index, HC = healthy control, qRT-PCR = quantitative real-time polymerase chain reaction, 16S rRNA = 16S ribosomal RNA, UC = ulcerative colitis.

*E faecalis* was the most common bacterial species of the 4 pathogens detected in all IBD cases, occurring in 95.74% (N = 45/47) of patients with UC and 86.57% (N = 58/67) of patients with CD (Table 3). A significant increase in *E faecalis* levels appeared to be associated with clinically active disease in patients with CD (Fig. 2A). Accordingly, we found a statistically significant and positive relationship between high-level *E faecalis* colonization and CDAI score (*R* = 0.3118, *P* = 0.0108, Fig. 2B, Table 4). Furthermore, in both UC and CD, high-level *E faecalis* colonization was significantly associated with increased FC (*P* = 0.002 in UC; *P* = 0.016 in CD).

**Table 3:**
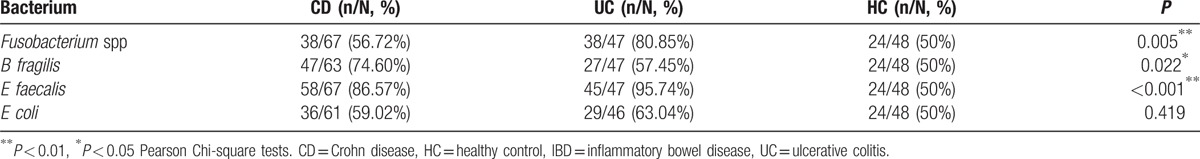
Frequency of IBD patients with bacterial colonization.

**Table 4:**
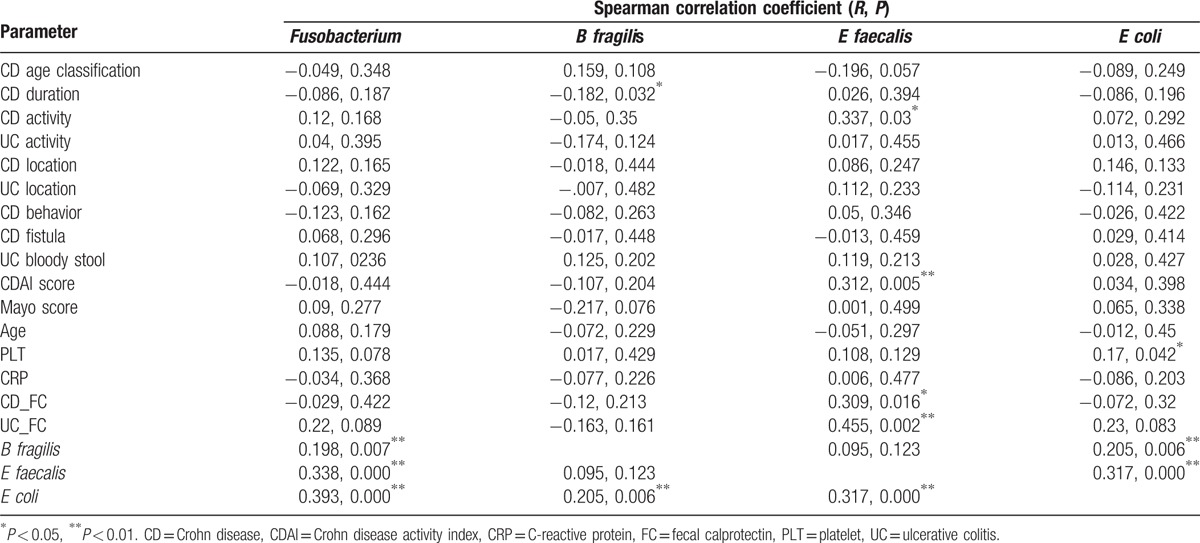
Correlations between bacterial populations and clinical parameters.

Infection with *Fusobacterium* spp was also frequently observed in patients with UC (80.85%, 38/47; Table 3). We analyzed the correlation between *Fusobacterium* spp levels and *E faecalis*, *B fragilis*, and *E coli* levels, and found marked associations between all bacterial communities (*Fusobacterium* spp vs *B fragilis*, *P* = 0.007; *Fusobacterium* spp vs *E faecalis*, *P* < 0.0001; *Fusobacterium* spp vs *E coli*, *P* < 0.0001; Table 4).

## 4 Discussion

IBD is a multifaceted and heterogeneous disease, and increasing evidence supports a significant role for the gut microbiome in promoting IBD pathogenesis. Efforts have been made to explore the possible pathogens involved in IBD development, but it is still unclear which specific pathogen or combination of agents are responsible for triggering or enhancing IBD. In this study, we quantified the levels of pathogenic microbes known to be associated with CRC in a Chinese patient cohort to identify a link between bacterial composition and IBD severity.

New pathogens are continuously being discovered.^[36]^ One of the most robust associations between gut bacterial content and CRC has been demonstrated for *Fusobacterium* spp, a heterogeneous oral pathogen that is also a common resident of the human gut mucosa.^[37]^ The precise mechanisms by which *Fusobacterium* spp promotes CRC are not fully understood; however, many studies have provided insight into the role of this pathogen in CRC. As a mucosal adherent bacterium, *Fusobacterium* spp is located proximal to host cells, and augments CRC risk primarily through direct interaction with the host.^[38–41]^ Strauss et al^[42]^ found that the invasive potential of gut mucosa-derived *Fusobacterium nucleatum* positively correlated with IBD status of the host. In our study, we also found that *Fusobacterium* spp levels were significantly increased in the feces of patients with IBD, especially in patients with UC. These findings in Chinese patients are consistent with important reports from Strauss et al, suggesting that the association between *Fusobacterium* spp infection and IBD development is unaffected by geographic and ethnic factors. Collectively, these studies highlight the importance of reducing the risk of CRC in IBD patients with high-level *Fusobacterium* spp colonization.

*E faecalis* is a common opportunistic pathogen found in the alimentary tract of both humans and animals that can trigger IBD.^[43]^ Our study reveals that increased *E faecalis* is a prominent feature in patients with IBD, especially CD. A significant increase in *E faecalis* levels appeared to be associated with clinically active disease in patients with CD. Accordingly, we found that high-level *E faecalis* colonization had a significant, positive relationship with CDAI score as well as with FC levels. Elevated FC is a predictor of relapse and clinically active disease in patients with IBD.^[44,45]^ To our knowledge, this report is the 1st to demonstrate that *E faecalis* infection is positively associated with clinically active CD. However, studies with more participants will be required to further substantiate these findings.

Swidsinski et al^[46]^ discovered that *B fragilis* biofilm is the main feature of IBD. Some studies have indicated that ETBF could be at the origin of the disease, but its presence is at least associated with active disease and relapse.^[47,48]^ This association may potentially be attributed to the ability of fragilysin to diminish epithelial barrier function, increase bacterial internalization, and enhance antibody responses.^[49]^ The barrier function of some IBD patients is abnormal, and organisms penetrating the lamina propria can initiate an immunologic overreaction and disease onset.^[50,51]^ Therefore, colonization by an organism such as ETBF, which can further reduce barrier function, may very well be linked to disease relapse. Moreover, experiments in mice using a dextran sulfate-induced model of colitis showed that fragilysin induced a greater degree of inflammation and more severe disease in the presence of ETBF compared to nonenterotoxigenic *B fragilis* (NTBF).^[52]^ In our patient cohort, we found that *B fragilis* was more enriched in patients with CD than in patients with UC, but its presence was not associated with active disease or relapse. This may be explained by the interindividual variation in *B fragilis* levels observed in our population, in which the pathogen either could not be detected at all in a few patients or was detected at high levels (l0^7^ copies/g feces) in others. Therefore, this finding also supports the notion that the composition of the human gut microbiome is influenced by geographic and ethnic factors.^[53,54]^

It has been previously shown that mucosa-associated *E coli* is increased in patients with CD^[36,55–59]^ and CRC,^[57,60]^ and to a lesser extent in those with UC.^[31,32,61]^ Prorok-Hamon et al^[33]^ found that IBD and CRC share in common a colonic mucosal *E coli* that expresses genes relevant to pathogenic processes, including M-cell translocation, angiogenesis, and genotoxicity. In our study, we did not detect significant differences in the total number of adherent *E coli* between IBD patients and controls or between CD and UC. This inconsistency may be due in part to the method by which *E coli* was quantified; the aforementioned studies were performed by using intestinal biopsies, which yield higher numbers of intestinal organisms that associate with the mucosal surface and harbor properties that influence the host.

In our further analysis of different combinations of bacterial colonization/infection, we found that the combination of *Fusobacterium* spp and *E faecalis* was prominent (80.85%) in UC whereas the combination of *B fragilis* and *E faecalis* was prominent (65.08%) in CD. These findings support the notion that there may not be a particular single bacterium responsible for the progression of diseases such as IBD and CRC. On the contrary, it could be that disease progression is determined by the interaction of a variety of coexisting bacteria. Indeed, studies of gut microbiota with the 16S rRNA gene sequencing method have suggested that IBD is associated with reduced biodiversity, decreased abundance of several taxa in the Firmicutes phylum, and increased abundance of *Gammaproteobacteria*.^[34,62]^

There are some limitations to our study. First, the implementation of an observational study design cannot untangle the causal relationship between the gut microbiome and IBD. Samples were collected from patients already diagnosed with IBD and therefore, whether infection with *Fusobacterium* spp or *E faecalis* causes IBD or represents a consequence of disease must be investigated in future studies. If a causative link is proven, antibiotics or vaccines may be used as potential therapies to treat or prevent IBD. Second, the sample size of the healthy control group was rather small. In addition, due to the lack of follow-up data, we could not evaluate any associations between these bacteria and a longer time-to-relapse. Furthermore, it is plausible that the presence of the pathogens examined in fecal samples may not accurately reflect the microbiome dynamics in the actual gut. Indeed, adherent bacteria may exert greater effects on gene expression in colon mucosal cells than transient bacteria that are flushed in fecal samples. Additional studies are needed to determine the mechanisms by which *E faecalis*, *Fusobacterium* spp, and *B fragilis* contribute to pathogenesis, infiltrate the gastrointestinal tract during chronic inflammation, and confer resistance to therapy. Finally, larger studies encompassing stool and colon tissue samples across different stages of IBD development are required to identify and establish strong bacterial markers of IBD.

## Acknowledgments

The authors thank National Clinical Key Institute Foundation of the Chinese Health and Family Planning Ministry (Grant No. 2013-544), the National Natural Science Foundation of China (81372633), and Guangzhou Medical and Technology Project (2014A011010041) for the support.
